# Single-cell profiles reveal tumor cell heterogeneity and immunosuppressive microenvironment in Waldenström macroglobulinemia

**DOI:** 10.1186/s12967-022-03798-6

**Published:** 2022-12-09

**Authors:** Hao Sun, Teng Fang, Tingyu Wang, Zhen Yu, Lixin Gong, Xiaojing Wei, Huijun Wang, Yi He, Lanting Liu, Yuting Yan, Weiwei Sui, Yan Xu, Shuhua Yi, Lugui Qiu, Mu Hao

**Affiliations:** 1 State Key Laboratory of Experimental Hematology, National Clinical Research Center for Blood Diseases, Haihe Laboratory of Cell Ecosystem, Institute of Hematology & Blood Diseases Hospital, Chinese Academy of Medical Sciences & Peking Union Medical College, Tianjin, 300020 China; 2 Tianjin Institutes of Health Science, Tianjin, 301600 China

**Keywords:** Waldenström macroglobulinemia, Tumor cell heterogeneity, Cancer microenvironment, Immunotherapy

## Abstract

**Background:**

Waldenström macroglobulinemia (WM) is a rare and incurable indolent B-cell malignancy. The molecular pathogenesis and the role of immunosuppressive microenvironment in WM development are still incompletely understood.

**Methods:**

The multicellular ecosystem in bone marrow (BM) of WM were delineated by single-cell RNA-sequencing (scRNA-seq) and investigated the underlying molecular characteristics.

**Results:**

Our data uncovered the heterogeneity of malignant cells in WM, and investigated the kinetic co-evolution of WM and immune cells, which played pivotal roles in disease development and progression. Two novel subpopulations of malignant cells, CD19^+^CD3^+^ and CD138^+^CD3^+^, co-expressing T-cell marker genes were identified at single-cell resolution. Pseudotime-ordered analysis elucidated that CD19^+^CD3^+^ malignant cells presented at an early stage of WM-B cell differentiation. Colony formation assay further identified that CD19^+^CD3^+^ malignant cells acted as potential WM precursors. Based on the findings of T cell marker aberrant expressed on WM tumor cells, we speculate the long-time activation of tumor antigen-induced immunosuppressive microenvironment that is involved in the pathogenesis of WM. Therefore, our study further investigated the possible molecular mechanism of immune cell dysfunction. A precursor exhausted CD8-T cells and functional deletion of NK cells were identified in WM, and CD47 would be a potential therapeutic target to reverse the dysfunction of immune cells.

**Conclusions:**

Our study facilitates further understanding of the biological heterogeneity of tumor cells and immunosuppressive microenvironment in WM. These data may have implications for the development of novel immunotherapies, such as targeting pre-exhausted CD8-T cells in WM.

**Supplementary Information:**

The online version contains supplementary material available at 10.1186/s12967-022-03798-6.

## Background

Despite recent improvements in treatment strategies, including rituximab, proteasome inhibitors, bendamustine, and BTK-inhibitors, Waldenström macroglobulinemia (WM) remains an incurable disease [1, 2]. Our understanding of the pathogenesis and immune escape mechanisms of WM is still limited [3]. The clinical onset of WM is often characterized by the development of anemia and progressive tumor infiltration, highlighting the importance of tumor infiltration in disease development and progression [4]. Genetic analyses have demonstrated recurrent mutations of the myeloid differentiation primary response-88 (MYD88) gene in more than 87.5% of WM patients in our lymphoma center of Blood Disease Hospital, CAMS [5]. However, MYD88 mutations are neither specific nor sufficient for the pathogenesis of WM and can be detected in IgM monoclonal gammopathy of undetermined significance (MGUS) as well as in other B cell lymphomas [5–7]. The mechanisms underlying the pathogenesis as well as the cellular origin of WM remain poorly understood [8]. Recently, the immune microenvironment has emerged as a promising therapeutic target in hematologic malignancies [9], with the reciprocal influence underlying the co-evolution of tumor cells and immune cells. However, studies on the immune microenvironment in WM are currently limited to the evaluation of only a few parameters [10–12]. An in-depth exploration of the immune cell dysfunction and the co-evolutionary with WM cells may reveal opportunities for effective intervention.

The aberrant T-cell marker expression on tumor cells of WM was revealed in our previous study [13]. However, the molecular characteristics and the underlying function of these WM cells have not been fully investigated. High-throughput single-cell RNA sequencing (scRNA-seq) is a powerful approach for studying heterogeneous tissues and kinetics cellular processes [14, 15]. Here, scRNA-seq analysis of BM mononuclear cells (BMNCs) from five newly diagnosed WM patients (NDWMs) and six healthy donors (HDs) were studied, and the heterogeneity of WM cells and non-malignant cells in the BM microenvironment was investigated. The aberrant expression of T cell markers was confirmed at the protein level in WM-B cells and WM-plasma cells by multicolor flow cytometry analysis. Pseudotime-ordered analysis elucidated that CD19^+^CD3^+^ malignant cells were presented at an early stage of B cell differentiation. Colony formation assay further identified that CD19^+^CD3^+^ malignant WM-B cells acted as potential WM precursors. Additionally, our results elucidated that the immunosuppressive states of T cells and dysregulated natural killer (NK) cells are highly correlated with WM cell infiltration in the BM microenvironment. Notably, exhausted cytotoxic CD8^+^ T cells with diverse stages were discriminated in the WM BM according to their distinct transcriptional characteristics. The precursor (pre-) exhausted CD8^+^ T cells with co-expression of exhaustion and cytotoxic markers were identified, and they were more responsive to immune therapies than the terminally exhausted CD8^+^ T cells. Altogether, our study facilitates further understanding of the biological heterogeneity of tumor cells and immunosuppressive microenvironment in WM. These data may have implications for the development of novel immunotherapies, such as targeting pre-exhausted CD8-T cells in WM.

## Materials and methods

### Sample collection

Fresh BM aspirates were collected, and BMNCs were isolated by ficoll density-gradient centrifugation. The baseline characteristics of 15 WM patients evaluated in this study are shown in Additional file 1: Table S1. This study was approved by the Institutional Ethics Review Boards from the Institute of Hematology and Blood Diseases Hospital, Chinese Academy of Medical Sciences. Written informed consents were obtained from patients and healthy donors before sample collection.

### scRNA-seq and data processing

3′-Biased 10× Genomics Chromium was applied for BMNCs. Sequencing reads were aligned against the GRCh38 human genome with STAR [16]. Seurat was used for data normalization, dimension reduction, clustering, and differential gene expression [17, 18]. The following criteria were applied to filter low-quality cells: gene number < 200 or > 6,000, UMI < 1000, ribosomal gene proportion > 0.4, or mitochondrial gene proportion > 0.1. A total of 44,770 cells passing the quality control were incorporated into the further analysis. The top 2000 variable genes were identified using the “vst” method in the Seurat “FindVariableFeatures” function. We constructed the cell-similarity relationship using the function “FindNeighbors” of Seurat with 30 principal component analysis (PCA) dimensions. The resolution was set to 0.5 to obtain a more refined result. The copy number variations (CNVs) of cells were inferred using inferCNV [19]. The cell lineage trajectory was inferred by Monocle2 and Monocle3 [20, 21].

### Cell function analysis

The gene set enrichment analysis (GSEA) was performed using the GSEA software [22, 23]. Ingenuity pathway analysis (IPA) was performed to characterize the biological functions of cells. Gene ontology (GO) and KEGG analysis were performed with cluster Profiler4 relying on genome-wide annotation packages (OrgDb) released by the Bioconductor project [24]. The gene signature scores were defined by AddModuleScore with Seurat. The used gene sets are listed in Additional file 1: Table S2. Proliferation index (PI) was calculated with the following equation: PI (%) = (S + G2M)/(G0/G1 + S + G2M) × 100%. Kappa^+^ cells or Lambda^+^ cells were defined with four genes’ expression (IGKC, IGLC2, IGLC3, and IGLC7) in each cell [25]. CellPhoneDB was used to estimate cell–cell interactions [26, 27].

### Bulk RNA-seq and data processing

cDNA libraries from CD19^+^CD3^+^ cells and CD19^+^CD3^−^ were sequenced according to the RNA-seq protocols. Transcript expression levels were quantified after normalizing the count data with the edgeR package [28].

### Flow cytometry analysis

Flow cytometry was performed on a CantoII flow cytometer, and the data were analyzed by Flowjo. Detailed information on the antibodies utilized is listed in Additional file 1: Table S3.

### Statistical analysis

Data analyses were performed with R language and GraphPad Prism 8.0 Software. Unpaired Wilcoxon tests and Chi-square tests were done between patients with low-tumor infiltration and HDs, and between low-tumor-infiltration patients and high-tumor-infiltration patients. Spearman’s correlation was applied to the correlation analysis between the tumor cell proportion and normalized T cell proportion in patients. Statistical significance was set at *P* < 0.05. *P < 0.05, **P < 0.01, and ***P < 0.001. Additional details are provided in Additional file 2: Methods S1.

## Results

### scRNA-seq analysis identified the kinetics interaction and co-evolution between malignant and non-malignant cells involved in the pathogenesis of WM

**Fig. 1:**
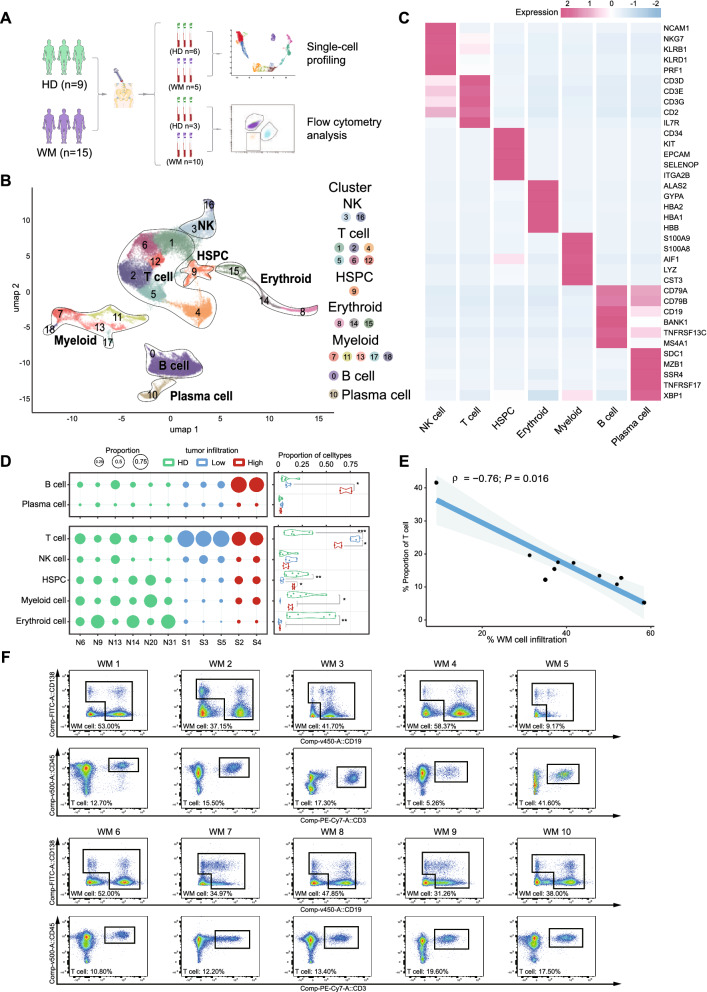
ScRNA-seq profiling and abnormality of erythroid development in WM. **A** Schematic representation of the experimental strategy based on 9 healthy donors (HDs) and 15 newly diagnosed WM patients. BMNCs from bone marrow aspirates of 5 WM patients and 6 HDs were measured by 10× Genomics-based scRNA-Seq. Another cohort including 3 HDs and 10 WM patients was applied to validate our findings by multicolor flow cytometry analysis. **B** UMAP plot showing the annotation and color codes for cell types of BMNCs from WM patients and HDs. **C** Heatmap showing the expression of marker genes in the indicated cell types. The bottom bars label the specific cell types. **D** Bubble plots showing the proportion of each cell type in each sample (left). Five WM patients were divided into low-tumor-infiltration group (blue) and high-tumor-infiltration group (red) according to the proportion of WM cells. Violin plots illustrating the fraction of each cell type in HDs, low-tumor-infiltration patients, and high-tumor-infiltration patients with unpaired Wilcoxon tests (right). The proportion of immune cells was calculated after normalizing the differential proportion of WM cells. **E** Fitted curve showing Spearman’s correlation between the fraction of WM cell infiltration and T cells’ proportion (normalizing differential WM cells) in another 10 WM patients. **F** Density dot plots of flow cytometry analysis displaying the population of T cells and WM cells derived from bone marrow aspirates of 10 WM patients

To delineate the multicellular in WM, a single-cell transcriptomics analysis was performed in BMNCs from WM patients (n = 5) and HDs (n = 6), and validated our findings by multicolor flow cytometry analysis in an additional cohort of three HDs and 10 NDWMs (Fig. 1A). A total of 44,770 cells passing the quality control stage were used for single-cell analysis (967–8761 cells; median: 3009 cells/patient), which generated a total of 4.13 billion mapped reads from which an average of 1460 genes (unique molecular identifier, UMI > 1) were detected (Additional file 3: Fig. S1A–C). Overall, 19 cellular clusters were annotated by performing differentially expressed genes (DEG) analysis and referring to canonical marker genes in the Cell Marker database (Fig. 1B). Based on the canonical marker gene expression, seven central cell populations were annotated: NK cells (NCAM1^+^), T cells (CD3D^+^), hematopoietic stem/progenitor cells (HSPCs, CD34^+^), erythroid cells (GYPA^+^), myeloid cells (LYZ^+^), B cells (CD19^+^, MS4A1^+^), and plasma cells (SDC1^+^, TNFRSF17^+^) (Fig. 1B, C). We found that these cell subtypes were present in both WM patients and HDs, albeit in different proportions (Additional file 3: Fig. S1D). Of note, among the five patients, our data showed that the B cell population was much higher in patients S2 and S4 compared with the other three patients (S1, S3, S5). However, these WM patients shared similar levels of plasma cell infiltration similar with HDs. Canonically, we regarded B cell and plasma cell population as WM cell infiltration in the following analysis. For the non-malignant cells, we found remarkable differences in immune cell population, such as T cells in normalized proportions between patients and HDs (Fig. 1D and Additional file 3: Fig. S1E). As imagined, the proportions of T cells significantly increased in WM BM milieu, especially in low WM-B cell infiltration ones (S1, S3, and S5) and vice versa (Fig. 1D). Correlation analysis consistently showed that the WM-B cell proportion notably negatively correlated with T cell proportion in BM of patients, which was further confirmed by flow cytometric analysis of 10 additional NDWM patients (Fig. 1E, F). These data provide direct evidence for the kinetics interaction and the co-evolution between tumor cells and immune cells in WM BM cellular ecosystem. Hence, our results support that the dysfunction of immune cells plays a pivotal role in the occurrence and progression of WM.

Anemia is a common symptom in WM patients, while the molecular mechanism has not been fully understood. Here, we noted that the proportions of HSPCs and erythroid cells were significantly decreased in WM BM milieu. We speculated anemia possibly resulted from the aberrant process of HSPC differentiation. To validate this hypothesis, we performed uniform manifold approximation and projection (UMAP) clustering of HSPCs and erythroid cells (Additional file 3: Fig. S1F). According to the expression of canonical marker genes (Additional file 3: Fig. S1G), three cell populations were annotated for HSPCs (CD34^+^, SPINK2^+^, CD52^+^), common myeloid progenitor (CMP) cells (GATA2^+^, ITGA2B^+^, CSF2RB^+^), and erythroid cells (GYPA^+^, ALAS2^+^, HBB^+^). Our data showed that the proportion of the erythroid cells in WM patients was lower than that in HDs (Additional file 3: Fig. S1H, unpaired Wilcoxon test for erythroid cells in HDs and WM patients, *P* < 0.05). Pseudotime analysis further indicated that the differentiation of HSPCs to erythroid cells was defected in WM microenvironment (Additional file 3: Fig. S1I). GO term analysis showed that the pathways of RNA splicing and ribonucleoprotein complex biogenesis were markedly enriched in HSPCs, supporting an aberrant RNA splicing process obstructed HSPC differentiation in WM patients (Additional file 3: Fig. S1J).

### Biological heterogeneity and novel tumor cell clusters were identified in WM at single-cell level

**Fig. 2:**
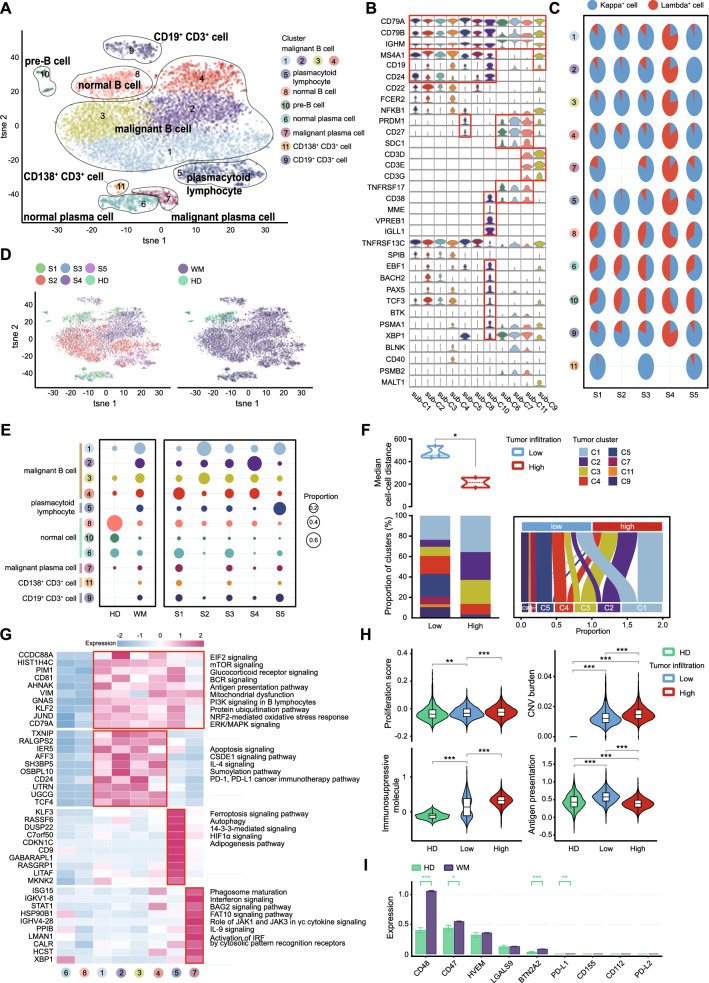
B cells, plasmacytoid lymphocytes, and plasma cells based on scRNA-Seq transcriptional profiles. **A** t-SNE plot showing the subtypes of B cells and plasma cells derived from HDs and WM patients. Each cluster is color-coded according to cell type. Cluster annotations are indicated in the figure. **B** Violin plots showing the expression of marker genes in each subcluster of B cells and plasma cells. **C** Pie charts showing the light chain restriction of each subcluster in 5 WM patients by color, Kappa+ cells (blue pies), and Lambda+ cells (red pies). **D** The t-SNE plots, showing cell origins by color, individual origin (left panel), and WM or HD origin (right panel). **E** Bubble plots showing the proportion of each subcluster in HDs and WM patients (left), and in each WM sample (right). **F** Violin plot showing the median of Euclidean distance between each cell of each individual in low-tumor infiltration patients, and high-tumor-infiltration patients (top). Stacked bar chart and Sankey diagram showing the proportions of each malignant subcluster in 2 groups (bottom). Statistical analysis is an unpaired Wilcoxon test between low-tumor-infiltration patients and high-tumor-infiltration patients. **G** Heatmap illustrating differential expressed genes (DEGs) between different types of malignant WM cells and their normal counterparts in WM (WilcoxDETest, *P* < 0.05), and their enriched pathways by Ingenuity Pathway Analysis (IPA, *P* < 0.05). **H** Violin plots showing the proliferation, copy number variation (CNV) burden, immunosuppressive molecule, and antigen presentation scores of normal B&Plasma cells from HD samples and malignant cells from low or high-tumor-infiltration patients. Unpaired Wilcoxon tests were done between patients with low-tumor infiltration and HDs, and between low-tumor-infiltration patients and high-tumor infiltration patients. **I** Bar charts showing the expression of immunosuppressive molecules in normal B&Plasma cells from HD samples and malignant cells from WM patients. Statistical analysis is an unpaired Wilcoxon test comparing WM patients and HDs. *P < 0.05, **P < 0.01, ***P < 0.001

To further investigate the WM tumor cell heterogeneity**,** we then focused on analyses of CD79^+^ cells according to *t*-distributed stochastic neighbor embedding (t-SNE) analysis that is annotated in Fig. 1C. Eleven subclusters were identified with high levels of CD79A, CD79B, and IGHM (Fig. 2A, B). Immunoglobulins (Ig) light chains expression patterns on B-cells and plasma cells were the hallmark of WM malignancies cells. For B cells and plasma cells in WM BM, the expression patterns of Ig light chain genes were further identified (Fig. 2C). According to the canonical mark gene expression and Ig light chain restriction analysis, sub-C1, sub-C2, sub-C3, sub-C4, sub-C5, sub-C8, and sub-C10 with high expressing of CD19 and MS4A1 were regarded as B cells. Among them, sub-C8 was annotated as normal B cells with a roughly 3:2 ratio of kappa^+^ to lambda^+^ cells. Sub-C1 to sub-C4 showed clonal Ig light chain restriction and then were designated as malignant B cells (WM-BC). Due to the combined high levels of PRDM1, CD19 and MS4A1, sub-C5 was recognized as malignant plasmacytoid lymphocytes. Of note, sub-C10 with higher levels of MME (CD10), VPREB1, and IGLL1 were regarded as pre-B cells, which existed predominantly in HDs. Sub-C6 and sub-C7 were plasma cells with high levels of SDC1 and TNFRSF17, while sub-C6 was normal plasma cells and sub-C7 was designated as malignant plasma cells (WM-PC) with clonal light chain restriction. Annotation of malignant or normal cells was validated by inferred copy number variation (CNV) scores, and CD19^+^ cells and CD138^+^ cells were compared separately with the control group (Additional file 3: Fig. S2A). Strikingly, our data demonstrated two novel malignant cell populations, sub-C9 (CD19^+^CD3^+^ cells) and sub-C11 (SDC1^+^CD3^+^ cells) with aberrant co-expression of T cell lineage markers (Fig. 2B, D). There were higher levels of sub-C7, sub-C6, sub-C9, and sub-C11 in low-tumor infiltration patients (S1, S3, and S5). Interestingly, we noted that WM-PC populations, sub-C7 (SDC1^+^TNFRSF17^+^) and sub-C11 (SDC1^+^CD3^+^), presented in low-tumor infiltration patients but were absent in high tumor infiltration ones. In addition, sub-C6 (normal plasma cells) was remarkably decreased in high-infiltration patients (Fig. 2E). Further analysis showed that patients with higher tumor infiltration had lower Euclidean cell–cell distance among the tumor cell sub-clusters than low-tumor infiltration patients (Fig. 2F). These findings indicate that tumor cell architecture involves in the malignant transformation, and the plasma cell differentiation was impaired in patients with higher malignant B cell infiltration.

To better characterize the biological features heterogeneity in diverse tumor cell subclones, we performed IPA analysis between malignant cells and their normal counterparts. Our results showed that the EIF2, mTOR, glucocorticoid receptor, BCR, PI3K, and protein ubiquitination pathways were remarkably enriched in WM malignant cells, including malignant B cells (sub-C1 to sub-C4), plasmacytoid lymphocyte (sub-C5) and malignant plasma cells (sub-C7, Fig. 2G). These findings may explain WM cell response to selective drug therapy including BTK inhibitors, steroids, and proteasome inhibitors. Additionally, we found that specific pathways were enriched in each type of malignant cell. CSDE1 gene, apoptosis, sumoylation (SUMO), and IL-4 signaling pathways were enriched in malignant B cells (sub-C1 to sub-C4). Ferroptosis, autophagy, 14-3-3-mediated signaling, and HIF1α pathways were enriched in malignant plasmacytoid lymphocytes (sub-C5). The immune-associated signaling pathways, including Interferon, phagosome maturation, BAG2, FAT10, JAK1, and JAK3 in γc cytokine signaling pathways were notably enriched in malignant plasma cells (sub-C7), suggesting that the WM-PC will be more susceptible to elimination by immune cells (Fig. 2G).

Since the immune escape process of tumor cells is mainly determined by the levels of immunosuppressive molecules, tumor antigen presentation ability, and tumor immunogenicity [29], we next evaluated the proliferation score, immunosuppressive molecule level, tumor copy number variations (CNVs), and the expression score of antigen processing and presenting machinery (APM)-related genes in WM cells. As expected, we found that the malignant cells in WM had higher proliferative, immunosuppressive signatures and CNV burden (Fig. 2H). The proliferation score, immunosuppressive score, and CNV burden were further elevated in patients with high tumor infiltration. The antigen presentation signature on tumor cells significantly decreased in high-tumor infiltration patients, and even was lower than that in HDs (Fig. 2H). Further analysis showed that malignant B cells (sub-C1 to sub-C4) displayed higher proliferation and the highest score of immunosuppressive molecules expression, and CNV burden, but the lowest antigen presentation signature (Additional file 3: Fig. S2B). These findings are consistent with the results shown above that malignant B cells (WM-BC), but not malignant plasma cells (WM-PC) present the survival advantage in WM. Among all the immunosuppressive genes, we noted the increased expressions of immune checkpoints CD47 and CD48 in WM (Fig. 2I), which may protect WM cells from avoiding immune cell attack. CD47 or CD48 would be the potential molecular targets of immune therapy in WM.

### Potential progenitor cell features of CD19^+^CD3^+^ WM cells were characterized in patients

**Fig. 3:**
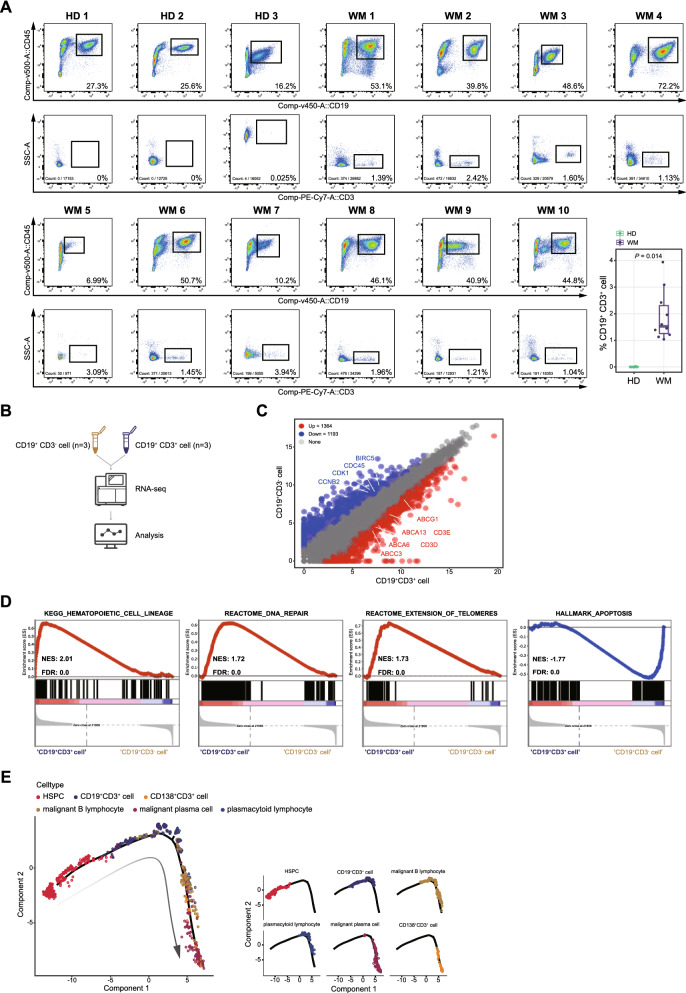
A novel tumor subcluster co-expressing CD19 and CD3, was identified as a potential progenitor of WM cells. **A** Density dot plots of flow cytometry analysis displaying the population of CD45^+^CD19^+^ cells and CD45^+^CD19^+^CD3^+^ cells derived from bone marrow aspirates of 3 healthy donors (HDs) and 10 WM patients. Boxplot showing the statistical result with unpaired Wilcoxon test (bottom right). **B** Flow chart of the strategy for RNA-seq of CD19^+^CD3^+^ cells and CD19^+^CD3^−^ cells in WM. **C** Volcano plot showing differential expressed genes (DEGs) between CD19^+^CD3^+^ cells and CD19^+^CD3^−^ cells in WM, with 1364 up-regulated and 1193 down-regulated. As to DEGs, the *P* and |log2 FC| were set at < 0.05 and > 1. **D** GSEA plots showing four enriched stemness-associated pathways of CD19^+^CD3^+^ cells compared with CD19^+^CD3^−^ cells in WM based on the RNA-seq of these two sorted cells. **E** Pseudotime-ordered analysis of HSPCs, CD19^+^CD3^+^ cells, CD138^+^CD3^+^ cells, and malignant WM cells from WM samples. Cell types are labeled by colors

In the above analysis, two novel populations of tumor cells with aberrant co-expression of T cell marker genes, including CD19^+^CD3^+^ (sub-C9) and CD138^+^CD3^+^ (sub-C11) were clearly identified, and were further validated by flow cytometry analysis in another cohort of 10 NDWM patients. The CD19^+^CD3^+^ and CD138^+^CD3^+^ malignant cells were presented in WM patient BM but absent in HDs (Fig. 3A and Additional file 3: Fig. S2C). As our data mentioned above, the sub-C9 were CD10 negative cells. Next, CD19^+^CD3^+^ cells were sorted by fluorescence-activated cell sorting (FACS), and examined the colony formation ability. Our data showed a relative higher colony formation capacity of CD19^+^CD3^+^ cells compared with CD19^+^CD3^−^ (Additional file 3: Fig. S2D). Bulk RNA-seq (Fig. 3B) analysis showed that multiple drug resistance-related genes, such as ABCG1, ABCA1, and ABCC3, were up-regulated, while cell cycle-related genes BIRC5, CDK1, and CCNB2 were down-regulated in CD19^+^CD3^+^ cells compared with CD19^+^CD3^−^ cells (Fig. 3C). GSEA analysis revealed that the DEGs of CD19^+^CD3^+^ cells were enriched in signal pathway of hematopoietic cell lineage, DNA repair, and extension of telomeres (Fig. 3D). Due to this small subset of WM-B cells co-expression T cell marker, we raised a presumption that the classical CD19^+^CD3^+^ cells would be de-differentiated from CD19^+^CD3^−^ cells during the process of disease development. To examine our hypothesis, we performed trajectory analysis using the Monocle2 algorithm. Classical WM cells (malignant B cells, malignant plasma cells, and malignant plasmacytoid lymphocytes), HSPCs, CD19^+^CD3^+^ cells, and CD138^+^CD3^+^ cells were included in the analysis. As Fig. 3E showed, CD19^+^CD3^+^ cells were notably enriched in the transition location downstream of HSPCs and upstream of malignant WM cells, while CD138^+^CD3^+^ cells were located at the terminal stage. These findings supported that the CD19^+^CD3^+^ cells could differentiate to classical WM cells as a progenitor of WM cell malignancy.

### WM BM displays suppressive natural killer cells and more exhausted T cells

**Fig. 4:**
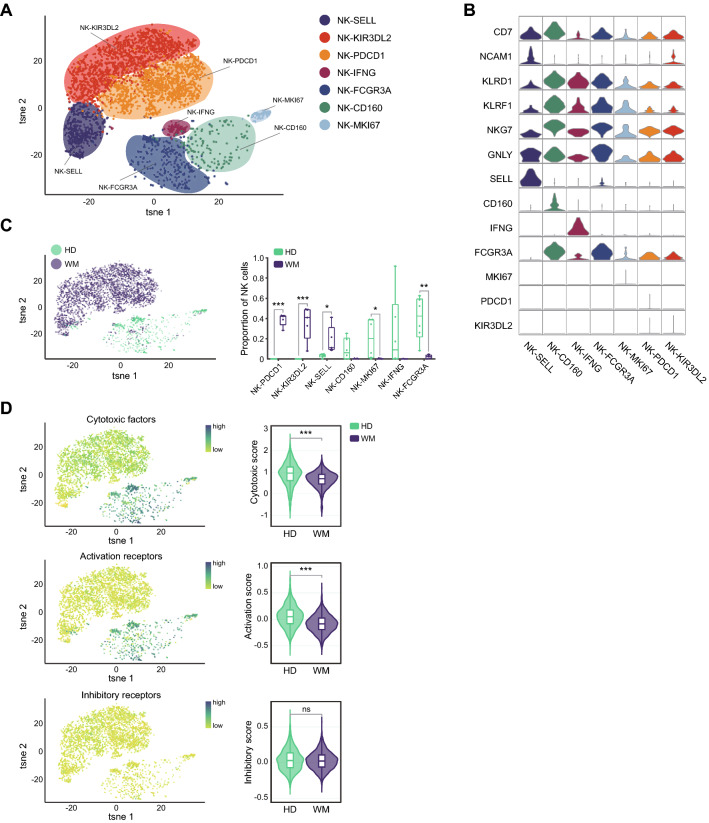
Distinct NK cells in WM from HD. **A** t-SNE projections of subclustered NK cells, labeled in different colors. Cell type annotations are provided in the figure. **B** Violin plots showing the expression of marker genes in each subtype of NK cells. **C** The t-SNE plots, showing the distinct distribution of NK cells in HD and WM (left). Boxplots illustrating the fraction of NK subtypes in HD and WM (right). Statistical analysis is an unpaired Wilcoxon test between HDs and WM patients. **D** t-SNE plots (left) and violin plots (right) illustrating the expression scores for genes related to cytotoxic factors, activating receptors, and inhibitory receptors of NK cells in HD and WM. Unpaired Wilcoxon tests were done between HDs and WM patients. ns: not significant, *P < 0.05, **P < 0.01, ***P < 0.001

Natural killer cell (NK) is a vital immune cytotoxicity cell in anti-tumor immunity. Our t-SNE analysis visualized seven NK cell subpopulations with high levels of the common NK cell markers CD7, NCAM1, KLRD1, KLRF1, and NKG7 (Fig. 4A, B). NK-SELL, NK-KIR3DL2, and NK-PDCD1 were mainly presented in WM patients, almost absent in HDs. However, NK-CD160, NK-FCGR3A, and NK-IFNG were mainly presented in HDs with high expression of cytotoxicity-associated genes including GZMK, GZMA, IFNG, and GZMB. NK-SELL cells were designated as naïve NK cells. NK-PDCD1 and NK-KIR3DL2 were identified as the immune-suppressive NK cells which were enriched in WM BM with overexpressing inhibitory-related genes HAVCR2 and PDCD1, and inhibitory KIRs (KIR3DL-2) (Fig. 4B, C). Further analysis showed that NK cells in WM exhibited a decreased cytotoxic and activation score, while the inhibitory score was similar between WM patients and HDs (Fig. 4D). This finding supported the suppressive function of NK cells in WM.

**Fig. 5:**
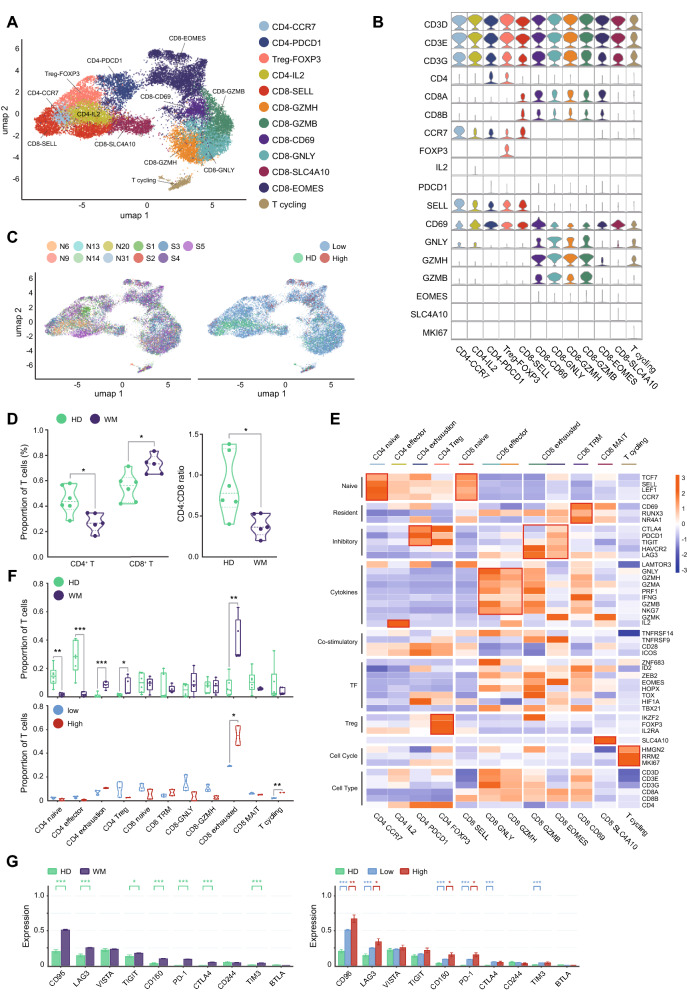
The subtypes of T cells in HD and WM. **A** t-SNE projections of subclustered T cells, labeled in different colors. Cell type annotations are provided in the figure. **B** Violin plots showing the expression of marker genes in each subtype of T cells. **C** The t-SNE plots, showing T cell origins by color, individual origin (left), and HD or low/high-tumor infiltration WM patients’ origin (right). **D** Violin plots showing the proportion of CD4+ and CD8+ T cells (left), and the ratio of CD4+ vs CD8+ T cells (right) in HD and WM samples. Statistical analysis is an unpaired Wilcoxon test between HDs and WM patients. **E** Heatmap indicating the expression of selected gene sets in T subtypes, including naive, resident, inhibitory, cytokines, co-stimulatory, transcriptional factors (TF), regulatory T (Treg) cell, cell cycle, and cell type. **F** Boxplots illustrating the fraction of T subtypes in HD and WM (top, unpaired Wilcoxon tests between HDs and WM patients), and in low/high-tumor-infiltration WM patients (bottom, unpaired Wilcoxon tests between low/high-tumor-infiltration WM patients), respectively. **G** Bar charts showing the gene expression of immune checkpoint receptors in CD8+ T cells of HD and WM (left, unpaired Wilcoxon tests between HDs and WM patients), and of low/high-tumor-infiltration WM patients (right, unpaired Wilcoxon tests between HDs and WM patients, and between low/high-tumor-infiltration WM patients). *P < 0.05, **P < 0.01, ***P < 0.001

For T cells, 12 subpopulations of T cells were further characterized according to the marker gene expression**.** Four clusters of CD4^+^ T cells (CD4-CCR7, CD4-PDCD1, CD4-FOXP3, and CD4-IL2), seven clusters of CD8^+^ T cells (CD8-SELL, CD8-GZMH, CD8-GZMB, CD8-CD69, CD8-GNLY, CD8-SLC4A10, and CD8-EOMES) and one cluster of cycling T cells were demonstrated (Fig. 5A, B). In general, all T cell subtypes were shared in WM patients and HDs. The increased infiltration of T cells was identified in WM patients compared with HDs. Consistently, we found a decreased infiltration of T cells in BM of high-tumor-infiltration patients compared with low-tumor-infiltration ones and HDs (Fig. 5C). These findings suggested that the immune escape of WM cells was caused by, at least in part, eliminating T cells in the microenvironment. Therefore, we compared the proportions of each T cell subtype between WM patients and HDs. The data showed that the fraction of CD8^+^ T cells was significantly increased in WM compared with HDs (72.4% versus 54.8% of total T cells). In contrast, the proportions of CD4^+^ T cells significantly decreased (27.6% versus 45.2% of total T cells). The CD4/CD8 ratio was significantly decreased in WM than HDs (Fig. 5D–F), which highlighted the disturbance of cellular immunity in patients. According to their characterized gene expression, four functional subpopulations of CD4-T cells and seven functional subpopulations of CD8-T cells were identified (Fig. 5E). For CD4^+^ subpopulations T cells, CD4 naïve (CD4-CCR7) cells, and CD4 effector (CD4-IL2) cells were significantly decreased, but CD4 exhaustion and CD4 regulatory T (Treg) cells were significantly increased (Fig. 5F). In addition, CD8 exhausted T cells were overwhelmingly elevated in WM, especially in high-tumor-infiltration ones (Fig. 5F). However, the proportion of CD4^+^ T cells did not differ between low- and high-tumor cell infiltration groups. CD8^+^ T cells in WM patients displayed higher levels of exhaustion markers including CD96, LAG3, TIGIT, CD160, PD-1, CTLA4, and TIM3. In high-tumor-infiltration patients, the levels of the exhaustion markers were further elevated on CD8^+^ cells compared with that in low-infiltration ones (Fig. 5G). Altogether, these results indicated that CD8^+^ T cell exhaustion states are exacerbated according to the elevation of WM cell infiltration.

### Pre-exhausted CD8^+^ T cells facilitate immunosuppressive BM and WM progression

**Fig. 6:**
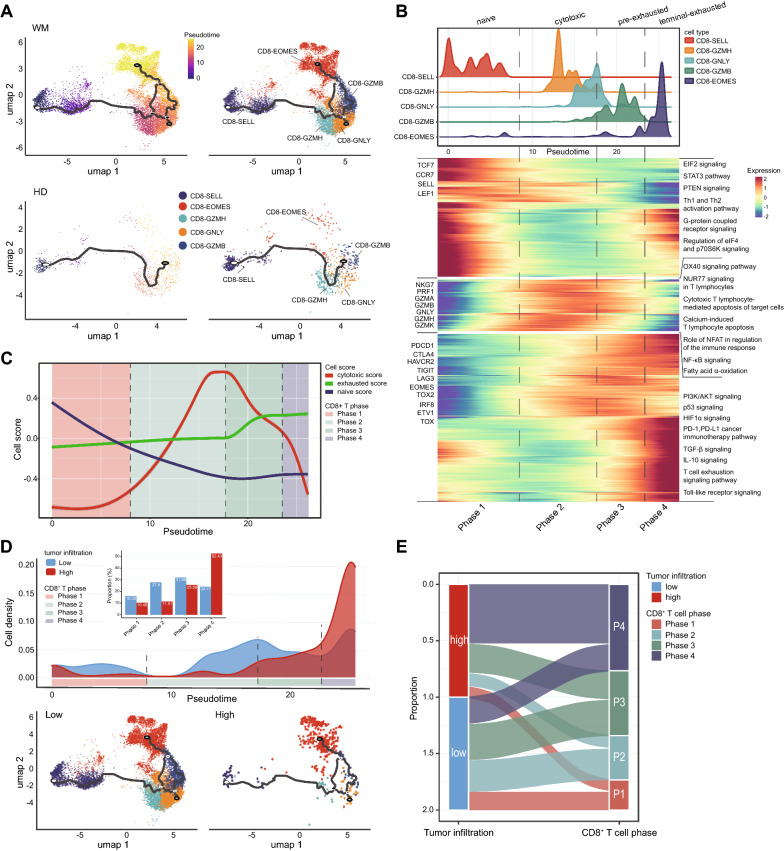
Analysis of CD8^+^ T cell transition states in HD and WM samples. **A** Pseudotime-ordered analysis of CD8^+^ T cells from HD (bottom) and WM (top) samples. CD8^+^ T cell subtypes are labeled by colors. **B** The distribution of CD8^+^ subtypes during the transition (divided into 4 phases), along with the pseudotime. Subtypes are labeled by colors (upper panel). Heatmap showing the dynamic changes in gene expression (divided into 3 groups) along the pseudotime, and their enriched pathways by Ingenuity Pathway Analysis (IPA, lower panel). **C** Two-dimensional plots showing the cell scores of genes related to naïve, cytotoxic, and exhausted CD8^+^ T cells along with the pseudotime (divided into 4 phases). Cells scores are labeled by line colors. **D** Density line plot showing the cell distribution in low/high-tumor-infiltration WM patients along with the pseudotime, by state (top). Histogram displaying the cell proportion of 4-phase CD8^+^ T cells in WM of 2 groups (top center, Chi-square test between low/high-tumor-infiltration WM patients, *P* < 0.001). 2D graph of the pseudotime-ordered CD8^+^ T cells, from WM of 2 groups (bottom). **E** Sankey diagram showing the proportions of 4-phase CD8^+^ T cells in low/high-tumor-infiltration WM patients

To further clarify the biological characteristics of the increasing exhausted CD8^+^ T cells in WM, we explored the kinetics of immune states of CD8^+^ T cells and cell transitions in WM by inferring the state trajectories using Monocle3. We excluded MAIT cells due to the distinct development processes relative to other CD8^+^ cells. As expected, naïve CD8 T cells (CD8-SELL) were located at the beginning of the trajectory path, whereas CD8 exhausted cells (CD8-GZMB and CD8-EOMES) were located at the terminal states in WM, and were absent in HDs (Fig. 6A). Of note, our further transcriptional variations investigation showed that the CD8^+^ T cell subclusters in WM could be obviously grouped for four different phases (Fig. 6B, C). Phase-1 CD8 T cells with the highest naive score and lowest cytotoxic score, were characterized by the upregulation of TCF7, CCR7, SELL, LEF1, and downregulation of GNLY, GZMA, and GZMH. Therefore, phase-1 CD8-T cells accounted for naive cells (Fig. 6B, C). IPA analysis indicated that EIF2, STAT3, and PTEN signal pathways were enriched in the phase-1 CD8^+^ T cells (Fig. 6B). Moreover, phase-1 CD8^+^ T cells exhibited the highest expression scores for the tricarboxylic acid (TCA) cycle and relatively lower expression scores for glycolysis. These observations indicated that phase-1 CD8^+^ T cells showed metabolic quiescence in which energy is produced via oxidative phosphorylation (OXPHOS) instead of aerobic glycolysis (Additional file 3: Fig. S2E). Phase-2 CD8^+^ T cells included CD8-GZMH and most CD8-GNLY cells, characterized by increasing levels of cytotoxic score. They were a bright expression of cytotoxic genes GZMA, GZMB, GNLY, and GZMH, but a weak expression of exhaustion markers (PDCD1, CTLA4, and HAVCR2) (Fig. 6B, C). The signal pathways analysis suggested that fatty acids represented a primary energy resource for effector T cells in phase 2.

Strikingly, we found that phase-3 and phase-4 CD8-T cells expressed elevated levels of exhaustion marker genes including PDCD1, CTLA4, HAVCR2, and TIGIT (Fig. 6B). In addition, the immune-suppressive pathways including TGF-β, IL-10, and T cell exhaustion were notably enriched in phase-3 and phase-4 cells. Of note, we further found the obvious differences between phase-3 and phase-4 exhaustion cells. As Fig. 6C showed, phase-3 cells co-expressed cytotoxic and exhausted genes. But phase-4 (CD8-EOMES) cells expressed the highest levels of exhausted genes (Fig. 6B, C). Therefore, we annotated phase-3 CD8-T cells were the pre-exhausted T cells. Phase-4 cells were the terminal exhausted T cells. Metabolic pathway analysis suggested that terminally exhausted cells exhibited lower levels of TCA cycle and glycolysis (Additional file 3: Fig. S2E), which contributes to reinforcing T cell exhaustion. Our results further supported that pre-exhausted T cells ultimately differentiated into terminally exhausted cells which lose the expression of TBX21 (T-bet), but up-regulation EOMES expression (Additional file 3: Fig. S2F). WM-specific CD8-T cell dysfunctionality is due to an inverse balance between T-bet and EOMES expression. The relative higher levels of phase-3 and phase-4, the exhausted states CD8^+^ T cells predominantly presented in WM patients (Additional file 3: Fig. S2G, Chi-square test, *P* < 0.0001).

For the influence of tumor cells on immunosuppressive milieu, the phase-4 exhausted CD8^+^ T cells remarkably elevated in high-tumor infiltration WM patients. Phase-1 naïve CD8^+^ T cells, phase-2 cytotoxic CD8^+^ T cells, and phase-3 pre-exhausted CD8^+^ T cells were predominantly distributed in low-tumor-infiltration samples (Additional file 3: Fig. S6D, E, Chi-square test, *P* < 0.001). Based on our knowledge, this is the first time reporting the diverse exhausted features of tumor infiltration cytotoxicity T cells in WM, which facilitated understanding the immunosuppressive BM milieu development and WM progression.

### CD47 is a potential immunotherapeutic target in rescuing T cell exhaustion and immune escape of WM

**Fig. 7:**
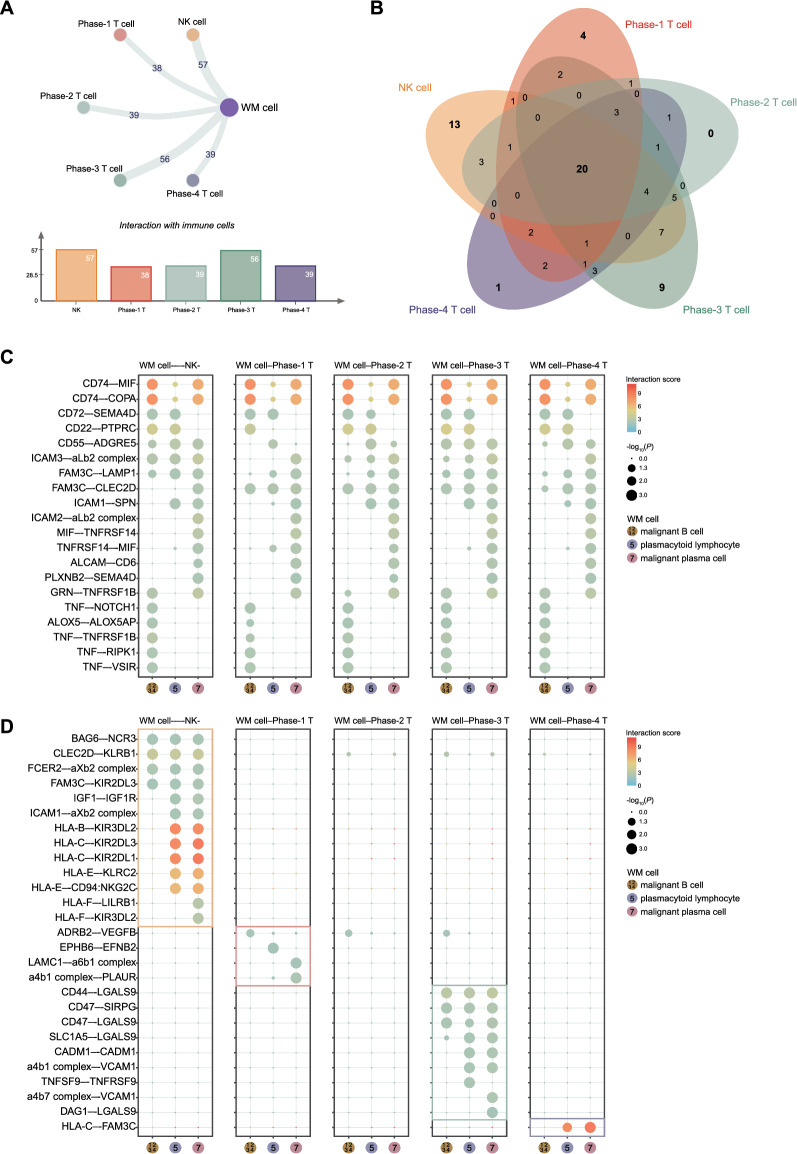
Crosstalk between malignant WM cells and immune cells in WM. **A** Network plot displaying the capacity for intercellular communication between malignant WM cells and NK, CD8^+^ T cells in 4 phases (top). The line thickness indicating the capacity for intercellular communication. The number of statistically significant ligand-receptor interactions between cell types are provided in the figure. Bar chart showing the number of significant ligand-receptor pairs in malignant WM cells and NK, CD8^+^ T cells in WM samples (bottom). Cell types are labeled by colors. **B** Venn plots illustrating the common and specific significant ligand-receptor pairs between malignant WM cells and NK, CD8^+^ T cells of 4 phases in WM patients. **C** Bubble plots showing molecular interaction states of 20 common ligand-receptor pairs (rows) between malignant WM cells and NK, CD8^+^ T cells in 4 phases (5 panels) in WM patients. Columns labeled by colors represent 3 main malignant WM cell subpopulations. **D** Bubble plots showing molecular interaction states of 27 ligand-receptor pairs (rows) specific to one immune cell between malignant WM cells and NK, CD8^+^ T cells in 4 phases (5 panels) in WM patients. Columns represent 3 main malignant WM cell subpopulations. Dot sizes and colors represent logarithmically transformed *P* values (Permutation test) and mean expression of interacting molecules in corresponding cell subsets. Molecules in big dots indicate that they were significant in WM cells or immune cells

The crosstalk between tumor cells and the immune system plays a vital role in the pathogenesis of malignancy. Our above findings have demonstrated that the T and NK cell dysfunction contributed to the immune escape of WM. Hence, we attempt to further reveal the molecular mechanism of the interactions between WM and immune cells, which will benefit the potential strategy development to rescue the immune suppression milieu in WM. Based on ligand-receptor analysis, we found 57 activated-ligand-receptor interaction pairs between WM and NK cells. Among the four phases of CD8-T cells, phase-3 pre-exhausted CD8^+^ T cells had 56 activated-ligand-receptor pairs interacting with WM cells, which presented closely connect with WM cells compared with the CD8^+^ T cells in the other three phases (Fig. 7A). Of note, twenty ligand-receptor pairs were common in the interaction of WM cells and immune cells, which involved in the immune escape of tumor cells (Fig. 7B). The top five ligand-receptor pairs, including CD74-MIF, CD74-COPA, CD72-SEMA4D, CD22-PTPRC, and CD55-ADGRE5, played critical roles in malignant B cells interacting with immune cells. CD74-MIF, CD74-COPA, and CD55-ADGRE5 were also involved in the interaction of malignant plasma cells and immune cells (Fig. 7C). Additionally, some specific ligand-receptor pairs were utilized in the interaction between WM cells and certain kinds of immune cells (Fig. 7D). Of note, we found that WM cells utilized specific chemokine interactions with phase-3 pre-exhausted CD8^+^ T cells via the CD47-LGALS9 and CD47-SIRPG axis (Fig. 7D), which explains the function of WM cells expressed a high level of CD47 (Fig. 2I). These findings suggested that CD47 molecule played important roles in the process of immune escape of WM via triggering CD8^+^ T cell exhaustion. CD47 would be a potential target in immune therapy, and targeting these ligand-receptor pairs would be efficiently rescued the immune escape in WM.

## Discussion

The opportunity to study entire transcriptomes in detail using scRNA-seq has fueled many important discoveries in the pathogenesis of malignancy, including WM [30]. Despite recent advances in the genetics of WM tumor cells, current information about the immune milieu in WM as well as the cell origin of this malignancy remain poorly understood [31]. There is a very high prevalence of MYD88^L265P^ in patients with WM, providing a genetic marker of the disease [32, 33]. However, many IgM MGUS patients with MYD88^L265P^ do not develop a B cell malignancy [34]. Progression from MYD88^L265P^ to malignant lymphoma may be fundamentally driven by the emergence of cooperating genetic alterations and the tumor microenvironment [35]. Our recent report indicates that MYD88^L265P^ is a recurrent mutation in WM patients in China [36], and all patients included in present study harbored MYD88^L265P^ mutation. Here, scRNA-seq analysis provides a comprehensive single-cell transcriptomic atlas to characterize cellular ecosystems in WM BM. We firstly delineated a novel model for the ecosystem of WM, wherein tumor cells and immune cells co-evolve kinetically, and clarified an aberrant immune suppressive milieu. These would provide novel insights into the disease development and progression.

Our previously published study reported the aberrant T-cell marker expression on tumor cells of WM by gene expression profiling [13]. The current study further confirms the finding of CD19^+^CD3^+^ malignant WM cells at single-cell resolution and validates at the protein level by flow cytometry analysis. Besides characterizing the three subgroups of canonical malignant cells in WM including B cells, plasma cells (PC), and plasmacytoid lymphocytes, two novel tumor cell subpopulations were identified at single-cell resolution with distinct transcriptomic profiles including CD19^+^CD3^+^ and CD138^+^CD3^+^ cells. This is the first time identified CD138^+^CD3^+^ tumor cells in WM. The CD138^+^CD3^+^ tumor cells also expressed high levels of IGHM, CD27, and XBP1, but were absent of CD19, CD22, CD24, and CD10 expression. Consistent with Catherine et al*.* recent report that MYD88^L265P^ mice exhibited very early increased IgM PC differentiation [37]. Continuous MYD88 activation is associated with expansion and the transformation of IgM-differentiating plasma cells. Additionally, our data demonstrated that canonical malignant plasma cells and CD138^+^CD3^+^ cells mainly presented in WM patients with low malignant B cell infiltration, and were almost absent in high-malignant B cell-infiltration ones. These findings support that a large number of malignant B cells will interfere with plasma cell differentiation in WM microenvironment, and further causes the de-differentiation of plasma cells. Tumor cell architecture involves in the malignant transformation, and the plasma cell differentiation was impaired in patients with higher malignant B cell infiltration.

In agreement with the flow cytometric pattern described previously, less mature CD138^+^PAX5^+^ plasma cells were significantly more abundant in WM than in marginal zone lymphoma (MZL) or plasma cell myeloma (multiple myeloma or MM). Conversely, more mature CD138^+^IRF4^+^ cells were rare in WM relative to MZL and myeloma [38]. Compared with malignant plasma cells (WM-PC), malignant B cells (WM-BC) present the survival advantage in WM. Therefore, our data support the notion that the optional treatment for WM is needed to rapidly eliminate malignant B cells [39].

CD3 and CD19 are the characteristic surface markers of mature T lymphocytes and B lymphocytes in human, respectively. Rizwan et al*.* recently reported a special subset of immune cells that characteristically dual expresses key lineage markers of both B and T cells (CD19^+^CD3^+^ cells) in type 1 diabetes patients, which proposes stimulating autologous CD4-T cells and may contribute to autoimmunity [40]. Additionally, CD19^+^CD3^+^ cells were also discovered in many types of cancer and would be a potential novel tumor immune marker as our previous study reported [13, 41]. Although the etiology of WM is unknown, recent research advances have all implicated autoimmune and chronic inflammatory conditions in the causation of the disease [42–44]. In this study, this malignant subpopulation was confirmed by the expressions of clonotypic kappa or lambda light chain/IGHM/CD22/CD27 and negative for CD10, CD24, and CD38 by scRNA-seq. This immunophenotype matches memory B cells (smIgM^−/+^/CD10^−^/CD19^+^/CD20^+^/CD27^+^/CD38^low+^/CD45^+^) [8]. Further analysis demonstrated that CD19^+^CD3^+^ malignant cells present an early stage of B cell differentiation by pseudotime analysis compared with CD19^+^CD3^−^ canonical B cells. Colony formation assay supported the progenitor cell features of CD19^+^CD3^+^ malignant cells. These findings propose that CD19^+^CD3^+^ cells may act as potential WM precursors. Tracing the cell of origin is one of the major directions of WM research [45, 46], as its identification would allow us to understand the development of the disease and uncover potential therapeutical targets [47]. Kaushal et al. recently reported that CD19^+^CD10^+^ pre-B cells and CD19^−^CD34^−^ pro-B cells harbor the phenotypic and molecular signatures of the malignant Waldenström clone [30]. We speculate the specific molecular signatures of WM, including MYD88 mutation, +4, 6q-, +12, and +18q would be already harbored in CD19^+^CD3^+^ cells, the potential progenitor cells of WM. Due to the small proportion of CD19^+^CD3^+^ and CD138^+^CD3^+^ among malignant cells, the cell origin and the pathophysiological functions including MYD88 state of them were not analyzed in the present study, which would be further investigated in our future study.

Cancer immune evasion is a major stumbling block in designing effective anticancer therapeutic strategies [48]. Consistent with previous report by Beltra et al. we identified four exhausted phases of CD8^+^ T cells, especially the precursor exhausted CD8^+^ T cells in the immunosuppressive microenvironment of WM for the first time [49]. Understanding the features of and pathways to T cell exhaustion has crucial implications for the success of checkpoint blockade therapies [50]. Kallies et al. recently reported that these precursor exhausted T cells are responsible for the proliferative burst that generates effector T cells in response to immune checkpoint blockade targeting programmed cell death 1 (PD1), and increased pre-exhausted cell frequencies have recently been linked to increased patient survival [51]. Our results showed malignant cells highly expressed immune checkpoint molecules CD47 and CD48, and strongly interacted with pre-exhausted T cells via CD47-LGALS9 and CD47-SIRPG molecules. Since pre-exhausted T cells re-engaged some effector biology and increased in response to immune checkpoint blockade [49, 52, 53], our results support that CD47 would be a potential therapeutic target to reverse CD8^+^ T cells cytotoxic dysfunction in WM. Much progress including ongoing clinical trials has been made in targeting CD47 for cancer immunotherapy in solid tumors and hematological malignancies [54–56]. Since low-tumor-infiltration patients displayed a high level of pre-exhausted CD8^+^ T cells compared to high-tumor-infiltration ones, we speculate that lower-tumor infiltration WM patients would respond well to immune therapies such as anti-PD-L1 antibodies according to Kallies et al. [51] report. However, CD8-EOMES T cells, the terminally exhausted cells, were resistant to reinvigoration by PD-L1 blockade [49, 52, 53]. Our data demonstrated that metabolic reprogramming of terminally exhausted CD8^+^ T cells by IL-10 efficiently enhances anti-tumor immunity [57]. Of note, we found that terminally exhausted CD8^+^ T cells displayed a dysregulated metabolism, and the IL-10 signal pathway was notably enriched in them, indicating that reprogramming metabolic profiles may be essential for reactivating CD8^+^ T cells in WM. Therefore, these findings support the potential clinical value of IL-10-related therapy in WM, especially for patients with high tumor infiltration. In addition, WM malignant cells strongly interacted with NK and CD8^+^ T cells via the CD74-MIF axis. Blocking the CD74-MIF axis potentiates CD8^+^ T cell infiltration and drives pro-inflammatory M1 conversion of macrophages in the tumor microenvironment [58]. Thus, CD74 would be another potential therapeutic target for reversing the NK cell dysfunction in WM.

Although we described the aberrant status of immune cells in the WM microenvironment, including exhaustion of CD8^+^ T cells and functional depletion of NK cells, the fundamental mechanisms inherent to immune cell dysfunction need to be further clarified. Further studies are needed to unravel the underlying mechanisms and provide more potential strategies to reverse the immunosuppressive microenvironment in WM. This would help guide better-tailored therapy strategies and benefit the long-term controlling of WM.

In summary, in the present study, the biological heterogeneity of malignant cells and the altered dysfunctional states of immune cells were further uncovered in WM. This integrative analysis provides novel insights into the pathogenesis of WM and helps the development of novel therapies to benefit patients.

## Conclusions

Our study utilized single-cell RNA-sequencing and multicolor flow cytometry analysis to in-depth elucidate the cellular ecosystem of BM microenvironment of WM. The biological heterogeneity of malignant cells and aberrant immune cells was revealed. A novel tumor subcluster co-expressing CD19 and CD3 was identified as the tumor precursors of WM. We further elucidate the immunosuppressive states of T cells and natural killer cells according to WM cell infiltration. Of note, the precursor exhausted CD8^+^ T cells in WM were identified for the first time, which would elicit a more sensitive response to immune therapies than the terminally exhausted ones. This integrative analysis clarifies a comprehensive understanding of tumor cell heterogeneity and the altered functional states of immune cells in WM, which may have implications for the development of novel therapies.

## Supplementary Information

**Additional file 1: Table S1.** The baseline characteristics of WM patients. **Table S2.** Signature related gene sets used in this analysis. **Table S3.** Flow cytometric antibodies used in this study.

**Additional file 2: Methods S1.** Additional methods.

**Additional file 3: Figure S1.** Quality control and visualization of scRNA-seq data. **Figure S2.** Tumor heterogeneity and T cells state in WM.

## Data Availability

The datasets (raw data) generated in this study are available through the Genome Sequence Archive (GSA), BioProject ID: PRJCA013345, accession ID: HRA003473. Additional details are provided in Additional file 2: Methods S1.

## Abbreviations

WM: Waldenström macroglobulinemia
BM: Bone marrow
scRNA-seq: Single-cell RNA-sequencing
MYD88: Myeloid differentiation primary response-88
MGUS: Monoclonal gammopathy of undetermined significance
BMNCs: Bone marrow mononuclear cells
NDWMs: Newly diagnosed WM patients
HDs: Healthy donors
NK cells: Natural killer cells
CNVs: Copy number variations
GSEA: Gene set enrichment analysis
IPA: Ingenuity pathway analysis
GO: Gene ontology
PI: Proliferation index
UMI: Unique molecular identifier
DEGs: Differentially expressed genes
HSPCs: Hematopoietic stem/progenitor cells
UMAP: Uniform manifold approximation and projection
t-SNE: T-distributed stochastic neighbor embedding
Ig: Immunoglobulins
WM-BC: Malignant B cells
WM-PC: Malignant plasma cells
CSDE1: Cold shock domain containing E1
SUMO: Sumoylation
APM: Antigen processing and presenting machinery
FACS: Fluorescence-activated cell sorting
TF: Transcriptional factors
Treg: Regulatory T cell
TCA: Tricarboxylic acid
OXPHOS: Qxidative phosphorylation
PC: Plasma cells
MZL: Marginal zone lymphoma
MM: Plasma cell myeloma or multiple myeloma
PD1: Programmed cell death 1
