# Supplementary material for: Single-cell profiles reveal tumor cell heterogeneity and immunosuppressive microenvironment in Waldenström macroglobulinemia
Source: J Transl Med. 2022 Dec 9;20:576. doi: 10.1186/s12967-022-03798-6 (PMC9733185; doi:10.1186/s12967-022-03798-6)
Supplement: Supplementary file 1 — Additional file 1: Table S1. The baseline characteristics of WM patients. Table S2. Signature related gene sets used in this analysis. Table S3. Flow cytometric antibodies used in this study. [file 12967_2022_3798_MOESM1_ESM.pdf]

Additional file 1

Table S1 | The baseline characteristics of WM patients

Sample	Age	Gender	Stage of disease	MI type	IgM (g/L)	WBC (×10^9/L)	HB (g/L)	PLT (×10^9/L)	β2-MG (mg/L)	LDH (U/L)	MYD88 mutation	Tumor infiltration of used aspirate (%)
S1	43	M	ND	IgMκ	3.55	1.88	84	51	5.32	245	Positive	10.78
S2	55	M	ND	IgMκ	6.86	4.68	114	134	3.81	368.7	Positive	68.37
S3	48	M	ND	IgMκ	18.3	4.9	99	184	21.6	115.2	Positive	9.17
S4	47	M	ND	IgMλ	7.73	5.24	132	190	2.62	223.7	Positive	62.33
S5	58	M	ND	IgMκ	35.8	4.49	83	158	4.75	343	Positive	17.29
WM1	67	M	ND	IgMκ	54.2	3.23	70	86	6.83	101.1	Positive	53.0
WM2	55	M	ND	IgMκ	3.91	9.92	92	151	4.00	164.9	Negative	37.15
WM3	70	M	ND	IgMκ	13.0	8.17	107	192	4.13	486.8	Positive	41.7
WM4	76	M	ND	IgMλ	5.85	8.87	101	181	4.51	363.2	Positive	58.37
WM5	67	M	ND	IgMκ	11.1	3.82	107	120	11.4	108.3	Negative	9.17
WM6	74	M	ND	IgMκ	43.9	12.4	100	77	4.78	172.5	Positive	52.0
WM7	67	F	ND	IgMκ	59.7	6.96	86	159	13.0	188.6	Positive	34.97
WM8	62	M	ND	IgMκ	66.9	11.13	76	125	6.31	124.1	Positive	47.85
WM9	58	F	ND	IgMλ	38.6	10.4	106	284	3.39	148	Positive	31.26
WM10	80	M	ND	IgMκ	22.8	10.41	89	83	3.76	200.9	Positive	38.0

Abbreviations: M, male; ND, Newly diagnosed; MI, monoclonal immunoglobulin; Ig, immunoglobulin; WBC, white blood cell; HB, hemoglobin; PLT, platelet; β2-MG, beta 2-Microglobulin; LDH, lactate dehydrogenase.

Table S2 | Signature related gene sets used in this analysis

**Cell cycle**	**Immunosuppressive molecule**	**Antigen presentation**	**NK Cytotoxic**	**NK Activation**	**NK Inhibitory**	**TCA cycle**	**Glycolysis**
MAD2L2	TNFRSF14	ERAP1	FCGR3A	CD160	SIGLEC9	SDHB	ENO1
ZBTB17	CD48	ERAP2	GNLY	SLAMF6	PDCD1	PKLR	PKLR
E2F2	CD47	CANX	GZMK	CD84	TIGIT	SDHC	PFKFB2
SFN	CD80	HLA-A	GZMA	SLAMF7	HAVCR2	FH	HK2
HDAC1	CD86	HLA-C	TNF	LY9	KLRG1	MDH1	PFKFB4
CDC20	HLA-DRA	HLA-B	PRF1	CD244	KLRB1	SUCLG1	PPP2CA
CDKN2C	HLA-DRB1	TAP2	KLRD1	FCGR3A	KLRC1	IDH1	PPP2R5D
ORC1	CD274	PSMB8	IFNG	NCR3	CEACAM1	PDHB	PGK1
GADD45A	LGALS9	PSMB9	GZMH	KLRF1	SIGLEC7	SUCLG2	PPP2CB
CDC7	PVR	TAP1	GZMB	KLRK1	CD33	SDHA	PPP2R1B
CDC14A	NECTIN2	TAPBP	TYROBP	KLRC3	LAIR1	OGDH	PFKP
YWHAQ	BTN2A2	PSMB7	NKG7	KLRC1	LILRB1	MDH2	PFKFB3
BUB1	PDCD1LG2	PSMB5		CD226	KIR2DL3	DLD	HK1
ANAPC1		PDIA3		HCST	KIR2DL1	PDHA1	PGAM1

MCM6		B2M		TYROBP	KIR3DL2	PGK1	GAPDH
ORC4		PSMB10		NCR1	KIR3DL1	IDH3G	TPI1
ORC2		PSMB6				ACO1	ENO2
CDC25A		CALR				PC	PFKM
GSK3B						DLAT	PKM
MCM2						SDHD	ALDOA
ANAPC13						PFKP	ENO3
STAG1						HK1	ALDOC
TFDP2						ENO2	GPI
ATR						PFKM	PPP2R1A
ANAPC4						CS	PFKL
MAD2L1						SUCLA2	HK3
CCNA2						PCK2	PGAM2
ANAPC10						DLST	GAPDHS
SKP2						PKM	PFKFB1
CCNB1						IDH3A	
CDK7						IDH2	
CCNH						ALDOA	
SKP1						ALDOC	
CDC23						ACLY	
CDC25C						IDH3B	
PTTG1						ACO2	
E2F3						PFKL	
CDKN1A						HK3	
CCND3						PGAM2	
MCM3						PCK1	
TTK						GAPDHS	
ORC3							
HDAC2							
MAD1L1							
YWHAG							
DBF4							
CDK6							
MCM7							
ORC5							
CUL1							
SMC1A							
STAG2							
PRKDC							
MCM4							
E2F5							
CCNE2							
YWHAZ							

RAD21							
MYC							
CDKN2A							
CDKN2B							
GADD45G							
CDC14B							
CDC26							
ABL1							
ANAPC2							
CDKN1C							
WEE1							
CCND1							
ATM							
CHEK1							
CDK1							
SMC3							
BUB3							
CCND2							
CDKN1B							
ESPL1							
CDK2							
CDK4							
MDM2							
ANAPC7							
ANAPC5							
RB1							
TFDP1							
CDC16							
TGFB3							
BUB1B							
CCNB2							
SMAD3							
PKMYT1							
CREBBP							
PLK1							
ORC6							
RBL2							
E2F4							
YWHAE							
TP53							
CDC6							
CDC27							
ANAPC11							

SMAD2							
SMAD4							
CDC25B							
PCNA							
E2F1							
RBL1							
YWHAB							
GADD45B							
FZR1							
CDKN2D							
CCNE1							
TGFB1							
CDC45							
CHEK2							
YWHAH							
MCM5							
RBX1							
EP300							
TGFB2							
PTTG2							
CCNB3							
SMC1B							
CCNA1							

Table S3 | Flow cytometric antibodies used in this study

**Antibodies**	**Source**	**Clone**	**Identifier**
Anti-human-CD45-V500	BD Biosciences	HI30	Cat. No. 560777
Anti-human-CD19-V450	BD Biosciences	HIB19	Cat. No. 560354
Anti-human-CD3-PE-Cy7	BioLegend	OKT3	Cat. No. 317334
Anti-human-CD138-FITC	BD Biosciences	MI15	Cat. No. 552723
