# Supplementary material for: Single-cell profiles reveal tumor cell heterogeneity and immunosuppressive microenvironment in Waldenström macroglobulinemia
Source: J Transl Med. 2022 Dec 9;20:576. doi: 10.1186/s12967-022-03798-6 (PMC9733185; doi:10.1186/s12967-022-03798-6)
Supplement: Supplementary file 2 — Additional file 2: Methods S1. Additional methods. [file 12967_2022_3798_MOESM2_ESM.pdf]

Additional file 2 | Methods S1. Additional methods.

Patient characteristics and scRNA-seq strategy

Bone marrow mononuclear cells (BMNCs) were obtained from 9 healthy donors (HDs) and 15 newly diagnosed WM patients. The clinical and biological characteristics of 15 WM patients are listed in **Suppl. Table 1.** This study was approved by the Institutional Ethics Review Boards of the Institute of Hematology and Blood Diseases Hospital, Chinese Academy of Medical Sciences (China). Written informed consent was obtained from patients and HDs before sample collection.

Sample collection and single cell preparation

BMMCs were isolated by Ficoll (MERCK) density-gradient centrifugation and cryopreserved at -80°C for less than five days until processed. The number and viability of cells was measured using a TC20 automated cell counter (Biorad). Dead cells (cell viability less than 80%) were removed by magnetic bead purification (Miltenyi Biotech) according to the manufacturer's protocol before scRNA-seq.

Single-cell RNA library preparation and sequencing

Chromium single-cell sequencing technology was performed following the manufacturer's protocol (10× Genomics). Library construction procedures were performed using the Chromium Single Cell 3' Library, Gel Bead & Multiplex Kit (10× Genomics, V2), strictly following the manufacturer's instructions. Then the cell suspensions were loaded onto the 10x Chromium Single Cell Controller to generate single-cell gel bead-in-emulsions (GEMs), and we performed barcoded reverse transcription of RNA within a single cell using a Verity Thermal Cycler (Life Technologies). Through reverse transcription in a single GEM, the barcodes were added to the RNAs released from lysed cells; then fragmentation, end repair, polyA tailing, and adaptor ligation were achieved according to the standard protocol. The cDNA purification and size selection were performed by SPRI select beads (Beckman Coulter), and the quality was evaluated using the Agilent Bioanalyzer. Finally, the libraries were sequenced on an MGISEQ-2000 sequencer as 150 bp paired-end reads by Beijing Genomics Institute (BGI, Shenzhen, China).

scRNA-seq data processing

The R package Seurat (version 3) was used for data normalization, scaling, dimensional reduction, clustering, differential expression analysis, and most visualizations [1,2]. The Cell Ranger Software Suite (version 3.0.2; 10x Genomics) was used to perform sample de-multiplexing, alignment, barcode processing, and unique molecular identifier (UMI) counting. Briefly, sequencing reads were aligned against the GRCh38 human reference genome with STAR, and count matrices were built from the resulting BAM

files [3]. Quality of cells was then assessed based on four metrics step by step: (i) The number of detected genes per cell; (ii) The number of detected UMI per cell; (iii) The proportion of mitochondrial gene counts; (iv) The proportion of rRNA genes counts (RNA18S5 or RNA28S5). The following criteria were then applied to filter low-quality cells: gene number < 200 or > 6,000, UMI < 1000, ribosomal gene proportion > 0.4 or mitochondrial gene proportion > 0.1. A total of 44,770 cells passing the quality control were incorporated into the further analysis. For the integration of the cells from different samples, the Gene-cell matrix of all samples was integrated with Seurat to remove batch effects across different samples. In parameter settings, the first 30 dimensions of principal component analysis (PCA) were used.

Dimensionality reduction, clustering of cells, and visualization

The filtered gene-cell matrix was first normalized using "LogNormalize" methods in Seurat v.3 with default parameters. The top 2,000 variable genes were then identified using the "vst" method in the Seurat "FindVariableFeatures" function. PCA was performed using the top 2,000 variable genes. Graph-based clustering was performed on the PCA-reduced data for clustering analysis with Seurat v.3. The resolution was set to 0.5 to obtain a more refined result. Briefly, the first 50 PCs of the integrated gene-cell matrix were used to construct a shared nearest-neighbor graph (SNN; "FindNeighbors" in Seurat), and this SNN was used to cluster the dataset ("FindClusters") using a graph-based modularity-optimization algorithm of the Louvain method for community detection. Then UMAP was performed on the top 30 principal components for visualizing the cells.

Cell cluster annotation with specific maker genes expression

"FindAllMarkers" in Seurat (Wilcoxon rank-sum test) was used to perform differential gene expression analysis. For each cluster, marker genes were generated relative to all other cells. Cluster annotation was confirmed using the R package SingleR, which compares the transcriptome of every single cell to reference datasets to determine cellular identity [4].

Single cell copy number variations (CNVs) calling

To identify malignant cells with clonal large-scale chromosomal CNVs, we used the inferCNV R package to infer the genetic profiles of each cell based on the average expression of large genes sets (101 genes) in each chromosomal region of the tumor genome compared to normal cells [5]. All B-cell-lineage cells of WM were input as interrogation group and normal B cells and plasma cells from HDs were sampled as control. Other parameters were set as default. For each cell, gene expression was re-standardized and values were limited as -1 to 1. The CNV score of each cell, namely mutation burden was calculated as

quadratic sum of CNV for each gene.

DEGs identification and functional enrichment analysis

Differential gene expression analysis between different sample groups within a cluster was performed using "FindMarkers" in Seurat (Wilcoxon rank-sum test). A gene was considered significantly differentially expressed if the false discovery rate (FDR) < 0.05. The heat map was then generated using the pheatmap R package for filtered DEGs. For pathway analysis, the C2 (curated gene sets) and C5 (ontology gene sets) were downloaded from the Molecular Signature Database (MSigDB, http://software.broadinstitute.org/gsea/msigdb/index.jsp). Gene set enrichment analysis (GSEA) was performed using the Java implementation of GSEA software (version 4.0) [6,7]. The Broad Institute Gene Set Enrichment Analysis website (www.broad.mit.edu/gsea) provides detailed information about the computational method.

Gene ontology (GO) enrichment analysis on DEGs was performed with R package cluster Profiler (v4.0.3), which supports statistical analysis and visualization of functional profiles for genes and gene clusters [8]. Terms with an adjusted p-value < 0.05 were considered as significantly enriched. Visualization of GO pathways of DEGs between IGHM$^+$ and conventional CD8$^+$ T cells was conducted with GOplot package [9]. The zscore indicates the up- or down-regulation of enriched pathways, which is calculated as follows: $zscore = \frac{(up-down)}{\sqrt{count}}$. Count is the number of genes assigned to a term. Whereas up and down are the number of assigned genes up-regulated (logFC>0) in the term or down- regulated (logFC<0), respectively.

In pathway enrichment analysis for the phase gene sets of CD8$^+$ T cell trajectory and DEGs between tumor subpopulations and their normal counterparts, Ingenuity Pathway Analysis (IPA) was performed to characterize the biological functions of cells. The differentially expressed genes between different cell types were uploaded into the IPA software for the core analysis, and identified the canonical pathways, diseases and functions, upstream regulators, and gene networks.

Calculating the proportion of Lambda$^+$ and Kappa$^+$ cells

The human genome has one kappa constant (IGKC) gene but variable number of lambda constants – IGLC1, IGLC2, IGLC3 and IGLC7 are functional isotypes. Let *t* be a chosen transcript count threshold and *K* the number of cells expressing at least t IGKC transcripts. Further let *L* be the number of cells expressing at least t transcripts of any of three out of the four functional lambda isotypes: IGLC2, IGLC3 and IGLC7 [10]. The IGLC1 transcript was not detected in any B cell, probably because complete overlap of its 3'end

with the IGLL5 gene precludes IGLC1 mRNA quantification with 3'end RNA-Seq assays. The % lambda$^+$ cells within a given population was calculated as: $Lambda^+ = \frac{L}{K+L}$. t = 1 was chosen. The estimated % of lambda$^+$ cells was robust to different values of *t* ranging between 1 and 5.

Cell function analysis based on scRNA-seq

The cell-cycle phase was computationally assigned for each individual cell through the "CellCycleScoring" function in Seurat. To determine the proliferation vitality of tumor cells, we labeled each WM subcluster with a proliferation index (PI) based on the cell-cycle phase using the following equation: $PI\ (\%) = \frac{(S+G2M)}{(\frac{G0}{G1}+S+G2M)} \times 100\%$. We used cell scores to evaluate the degree to which individual cells expressed a certain predefined expression gene set using the "AddModuleScore" function in Seurat with default settings. To define metabolism phenotypes in CD8$^+$ T cells in WM and HD, the metabolism signature determined as the mean expression of gene signatures involved in glycolysis and tricarboxylic acid (TCA) cycle were obtained from PathCards (https://pathcards.genecards.org/). The gene sets associated with the above functions were listed in **Suppl. Table 2**. Besides, we defined naïve, cytotoxic, and exhausted scores for CD8$^+$ T cells with 6 naive markers (SELL, CD28, LEF1, TCF7, CCR7, and S1PR1), 8 cytotoxicity associated genes (PRF1, IFNG, GNLY, NKG7, GZMB, GZMA, CST7, and TNFSF10), and 7 exhausted markers (CTLA4, HAVCR2, LAG3, PDCD1, CD96, CD160, and TIGIT). Cellular transcriptomic heterogeneity of samples was accessed by calculating the pair-wised Euclidean distance between single cells of the same sample.

Cell-cell interaction analysis

CellPhoneDB was used to estimate cell-cell interactions [11,12]. The interaction score refers to the total average of the mean expression value of a single ligand-receptor partner in the corresponding interacting cell type. The expression of any complex output by CellPhoneDB was calculated as the sum of the expression of the component genes.

Cell developmental trajectory

2D pseudotime-ordered analysis of HSPCs, CD19$^+$CD3$^+$ cells, CD138$^+$CD3$^+$ cells, and malignant WM cells was performed using Monocle2 [13]. The cell lineage trajectory of CD8$^+$ T was inferred by using Monocle3 [14]. We excluded MAIT cells according to their distinct development processes relative to other CD8$^+$ cells. After the cell trajectories were constructed, differentially expressed genes along the pseudotime were detected using the "differentialGeneTest" function.

Flow cytometry analysis

Fresh bone marrow aspirates obtained from WM patients and HDs after informed consent were placed in ethylenediaminetetraacetic acid (EDTA)-containing tubes and immediately transported to the lab. Fresh BMMCs were isolated by Ficoll density-gradient centrifugation and stained with fluorochrome-conjugated antibodies for 15 minutes at room temperature. Flow cytometry for BMNCs was performed on a FACSCanto II flow cytometer, and the data were analyzed using FlowJo V10 software. Details of the antibodies utilized are listed in **Suppl. Table 3**.

RNA sequencing and data processing

We sorted $CD19^{+}CD3^{+}$ cells and $CD19^{+}CD3^{-}$ cells from BMNCs by FACS AriaIII (BD). The RNA yield was determined by Nanodrop technology (Thermo Fisher Scientific), and quality was verified on the Agilent 2100 bioanalyzer (Agilent Technologies). cDNA libraries from $CD19^{+}CD3^{+}$ cells and $CD19^{+}CD3^{-}$ were sequenced according to the protocols for RNA-Seq. Briefly, the True Seq Stranded mRNA Sample Preparation Kit (Illumina) was used, followed by single-ended sequencing on the NextSeq500 Sequencing System (Illumina) with a read length of 75 bp. Raw reads were pre-processed using Fast QC software. PCR duplicates, reads that only contain adapter, ploy-N, and reads with low quality (score ≤ 5) were removed. Clean reads were then used for subsequent analyses. RNA-seq reads were aligned to GRCh38 using STAR v.2.4.2a and quantified on Gencode v.24. Transcript expression levels were quantified after normalizing the count data with the edgeR package [15]. Only genes with P-value < 0.05 and | log2(Fold change) | > 1 are considered differentially expressed genes.

Colony-formation assay

A total of 1,000 $CD19^{+}CD3^{+}$ cells and 1,000 $CD19^{+}CD3^{-}$ cells were separately plated in triplicate in 1.1 ml methylcellulose-based medium (MethoCult™ H4100, StemCell Technologies) per 12-well and incubated for 3-4 weeks, replenishing with 10% FBS cell culture medium twice a week. The culture plate was taken photo both under inverted microscope and high-content analysis system (Operetta CLS). Colonies consisting of more than 40 cells were scored.

Statistical analysis

Data analyses were performed with R language and GraphPad Prism 8.0 Software. Statistical significance was set at $P < 0.05$. *$P < 0.05$, **$P < 0.01$, and ***$P < 0.001$.

References

1. Butler A, Hoffman P, Smibert P, Papalexi E, Satija R. Integrating single-cell transcriptomic data across different conditions, technologies, and species. *Nat Biotechnol*. 2018;36(5):411-420.
2. Stuart T, Butler A, Hoffman P, et al. Comprehensive Integration of Single-Cell Data. *Cell*. 2019;177(7):1888-1902.e1821.
3. Dobin A, Davis CA, Schlesinger F, et al. STAR: ultrafast universal RNA-seq aligner. *Bioinformatics*. 2013;29(1):15-21.
4. Aran D, Looney AP, Liu L, et al. Reference-based analysis of lung single-cell sequencing reveals a transitional profibrotic macrophage. *Nat Immunol*. 2019;20(2):163-172.
5. Patel AP, Tirosh I, Trombetta JJ, et al. Single-cell RNA-seq highlights intratumoral heterogeneity in primary glioblastoma. *Science*. 2014;344(6190):1396-1401.
6. Subramanian A, Tamayo P, Mootha VK, et al. Gene set enrichment analysis: a knowledge-based approach for interpreting genome-wide expression profiles. *Proc Natl Acad Sci U S A*. 2005;102(43):15545-15550.
7. Mootha VK, Lindgren CM, Eriksson KF, et al. PGC-1alpha-responsive genes involved in oxidative phosphorylation are coordinately downregulated in human diabetes. *Nat Genet*. 2003;34(3):267-273.
8. Wu T, Hu E, Xu S, et al. clusterProfiler 4.0: A universal enrichment tool for interpreting omics data. *Innovation (N Y)*. 2021;2(3):100141.
9. Walter W, Sánchez-Cabo F, Ricote M. GOplot: an R package for visually combining expression data with functional analysis. *Bioinformatics*. 2015;31(17):2912-2914.
10. Rai A, Greening DW, Chen M, Xu R, Ji H, Simpson RJ. Exosomes Derived from Human Primary and Metastatic Colorectal Cancer Cells Contribute to Functional Heterogeneity of Activated Fibroblasts by Reprogramming Their Proteome. *Proteomics*. 2019;19(8):e1800148.
11. Vento-Tormo R, Efremova M, Botting RA, et al. Single-cell reconstruction of the early maternal-fetal interface in humans. *Nature*. 2018;563(7731):347-353.
12. Efremova M, Vento-Tormo M, Teichmann SA, Vento-Tormo R. CellPhoneDB: inferring cell-cell communication from combined expression of multi-subunit ligand-receptor complexes. *Nat Protoc*. 2020;15(4):1484-1506.
13. Qiu X, Mao Q, Tang Y, et al. Reversed graph embedding resolves complex single-cell trajectories. *Nat Methods*. 2017;14(10):979-982.
14. Tambalo M, Mitter R, Wilkinson DG. A single cell transcriptome atlas of the developing zebrafish hindbrain. *Development*. 2020;147(6).
15. Robinson MD, McCarthy DJ, Smyth GK. edgeR: a Bioconductor package for differential expression analysis of digital gene expression data. *Bioinformatics*. 2010;26(1):139-140.
