# Supplementary material for: Single-cell profiles reveal tumor cell heterogeneity and immunosuppressive microenvironment in Waldenström macroglobulinemia
Source: J Transl Med. 2022 Dec 9;20:576. doi: 10.1186/s12967-022-03798-6 (PMC9733185; doi:10.1186/s12967-022-03798-6)
Supplement: Supplementary file 3 — Additional file 3: Figure S1. Quality control and visualization of scRNA-seq data. Figure S2. Tumor heterogeneity and T cells state in WM. [file 12967_2022_3798_MOESM3_ESM.pdf]

Fig. S1

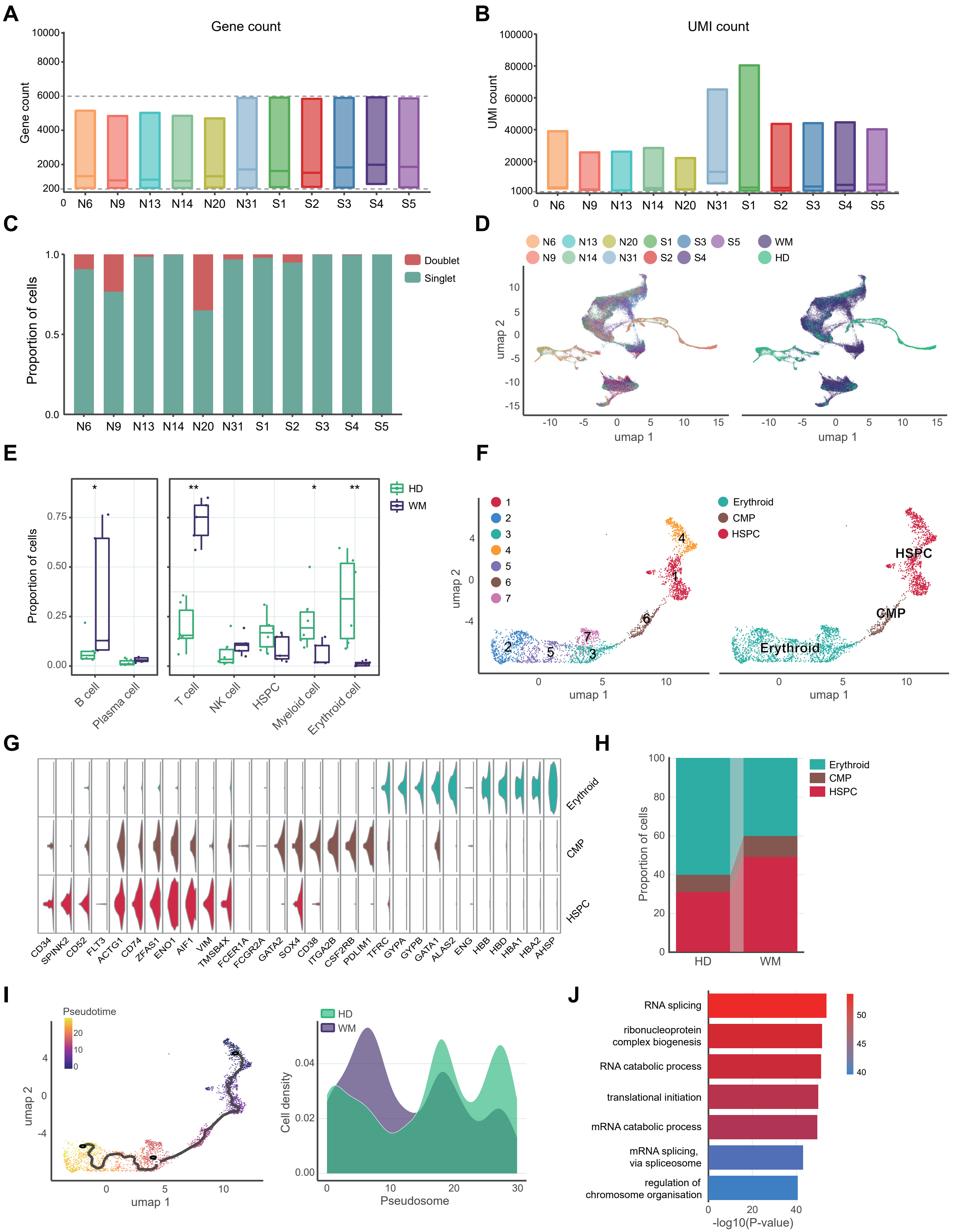

A
Gene count
Gene count
N6 N9 N13 N14 N20 N31 S1 S2 S3 S4 S5
B
UMI count
UMI count
C
Proportion of cells
Doublet
Singlet
D
umap 1
umap 2
WM
HD
E
Proportion of cells
HD
WM
B cell
Plasma cell
T cell
NK cell
HSPC
Myeloid cell
Erythroid cell
F
Erythroid
CMP
HSPC
umap 1
umap 2
G
CD34 SPINK2 CD52 FLT3 ACTG1 CD74 ZFAS1 ENO1 AIF1 VIM TMSB4X FCER1A FCGR2A GATA2 SOX4 CD38 ITGA2B CSF2RB PDLIM1 TFRC GYPA GYPB GATA1 ALAS2 ENG HBB HBD HBA1 HBA2 AHSP
Erythroid
CMP
HSPC
H
Proportion of cells
HD
WM
I
Pseudotime
umap 1
umap 2
Cell density
Pseudosome
J
RNA splicing
ribonucleoprotein complex biogenesis
RNA catabolic process
translational initiation
mRNA catabolic process
mRNA splicing, via spliceosome
regulation of chromosome organisation
-log10(P-value)

Fig. S1 | Quality control and visualization of scRNA-seq data

(A-B) Box plots showing filtering criteria for sequenced cells, including gene count and unique molecular identifier (UMI) count. The average values, maximum and minimum values of data after filtration are shown through the box plots.

(C) Bar charts showing the fraction of doublet cells filtered by DoubletDecon.

(D) UMAP plots showing cell origins by color, individual origin (left panel), and WM or HD origin (right panel).

(E) Boxplots illustrating the proportion of cells identified in BMNCs of HD and WM. The proportion of immune cells was calculated after normalizing the differential proportion of WM cells. *$P < 0.05$, **$P < 0.01$, unpaired Wilcoxon test between HDs and WM patients.

(F) UMAP projections of subclustered erythroid cells and HSPCs (left), and of cell type annotations (right), labeled in different colors.

(G) Violin plots showing the expression of marker genes in three cell subtypes.

(H) Stacked bar chart showing the proportions of each cell subtype in HD and WM, unpaired Wilcoxon test for erythroid cells in HDs and WM patients, $P<0.05$.

(I) UMAP plot of the pseudotime-ordered erythroid cells and HSPCs, from HD and WM (left). The cell density distribution, by cell origin, is shown at the right of the figure.

(J) Bar chart showing the enrichment of GO pathways, based on the DEGs of HSPCs between WM and HD.

Fig. S2

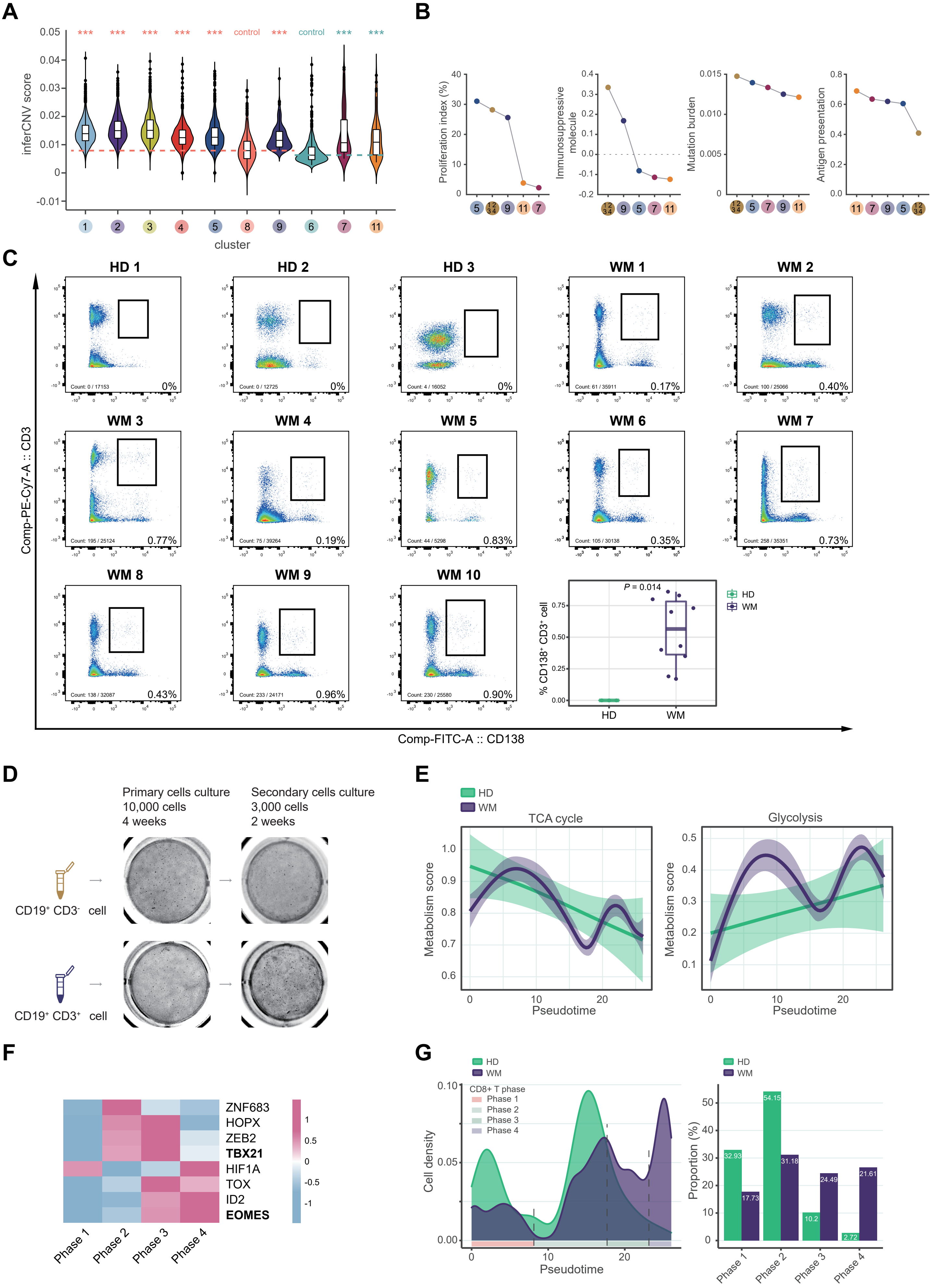

A
inferCNV score
cluster
*** *** *** *** *** control *** control *** ***
B
Proliferation index (%)
Immunosuppressive molecule
Mutation burden
Antigen presentation
C
HD 1
HD 2
HD 3
WM 1
WM 2
WM 3
WM 4
WM 5
WM 6
WM 7
WM 8
WM 9
WM 10
0%
0%
0%
0.17%
0.40%
0.77%
0.19%
0.83%
0.35%
0.73%
0.43%
0.96%
0.90%
Comp-PE-Cy7-A :: CD3
Comp-FITC-A :: CD138
P = 0.014
% CD138+ CD3+ cell
HD
WM
D
Primary cells culture
10,000 cells
4 weeks
Secondary cells culture
3,000 cells
2 weeks
CD19+ CD3- cell
CD19+ CD3+ cell
E
HD
WM
TCA cycle
Glycolysis
Metabolism score
Pseudotime
F
ZNF683
HOPX
ZEB2
TBX21
HIF1A
TOX
ID2
EOMES
Phase 1
Phase 2
Phase 3
Phase 4
G
HD
WM
CD8+ T phase
Phase 1
Phase 2
Phase 3
Phase 4
Cell density
Pseudotime
Proportion (%)
32.93
17.73
54.15
31.18
10.2
24.49
2.72
21.61
Phase 1
Phase 2
Phase 3
Phase 4

Fig. S2 | Tumor heterogeneity and T cells state in WM

(A) Violin plots showing the inferred copy number variation (CNV) scores among 10 subclusters of $CD79^+$ cells. $CD19^+$ cells and $CD138^+$ cells were compared separately with the control group by unpaired Wilcoxon test. ***$P < 0.001$.

(B) Line charts representing the median of proliferation, immunosuppressive molecule, mutation burden, and antigen presentation scores among 5 malignant WM subpopulations (sub-C1 to sub-C4, sub-C5, sub-C7, sub-C9, and sub-C11).

(C) Density dot plots of flow cytometry analysis displaying the population of $CD138^+CD3^+$ cells derived from bone marrow aspirates of 3 healthy donors (HDs) and 10 WM patients. Boxplot showing the statistical result with unpaired Wilcoxon test between HDs and WM patients (bottom right).

(D) Colony formation assay of $CD19^+CD3^+$ cells and $CD19^+CD3^-$ cells in WM.

(E) Two-dimensional plots showing the expression scores for genes related to the TCA cycle and glycolysis, in HD (green) and WM (purple) samples, along with the pseudotime.

(F) Heatmap indicating the expression of transcriptional factors (TF) in 4-phase $CD8^+$ T cells.

(G) Density line plot showing the cell distribution of HD and WM along with the pseudotime, by state (left). Histogram displaying the cell proportion of 4-phase $CD8^+$ T cells in HD and WM samples (right, Chi-square test between HDs and WM patients, $P < 0.001$).
